# Treatment Outcomes of Patients Placed on Treatment Under Directly Observed Therapy Short-Course (Dots)

**DOI:** 10.4103/0970-2113.44124

**Published:** 2008

**Authors:** Gurpreet Kaur, N.K. Goel, Dinesh Kumar, A.K. Janmeja, H.M. Swami, Meenu Kalia

**Affiliations:** Deptt. of Community Medicine, Govt. Medical College, Chandigarh, India

**Keywords:** DOTS, Cure rate

## Abstract

**Background:**

Tuberculosis continues to be a pressing health problem in India. The Revised National Tuberculosis Programme (RNTCP), an application of Directly Observed Treatment Short-course (DOTS) in India, launched in 1997 needs continuous evaluation.

**Objective:**

To study the outcomes of treatment among the patients put on DOTS under RNTCP in Chandigarh, UT.

**Material & Methods:**

A Longitudinal study was conducted during 2004-2005 in 13 Microscopic centres (MC's) spread over 2 Tuberculosis Units (TU's) under District Tuberculosis Centre (DTC) in Union Territory (UT), Chandigarh. A sample of 265 respondents, selected by two-stage stratified random sampling technique, was recruited in the study cohort. Data analysis was done using SPSS-10 statistical software package.

**Results:**

For Category I and Category II patients, the Success rate was 98.6% and 90.4% respectively. The overall default rate was 1.1% and failure rate was 2.6%. For re-treatment cases, failure rate was higher i.e. 5.8%. The sputum conversion rate among the new smear positive cases was 93.8% at 3 months of treatment. For the re-treatment cases, spu-tum conversion rate at 3 months was 94.1%.

**Conclusion:**

The study concludes that RNTCP is running successfully in UT Chandigarh, having high success rate and low default rate. The reasons for high failure rate should be explored in depth.

## INTRODUCTION

Tuberculosis remains the major cause of death from a single infectious agent among adults in developing countries. It is estimated that about 8 million individuals develop new clinical disease and 3 million people die of tuberculosis each year.^1^ India alone accounts for approximately 1/5^th^ of total global tuberculosis incidence.^2^ Every year 1.8 million new cases occur in India of which 0.8 million are infectious.^2^

Recognizing that tuberculosis epidemic is out of control in many parts of the world, the WHO declared tuberculosis to be a global health emergency in April 1993. It developed a five point strategy known as Directly Observed Treatment Short-course (DOTS) in order to combat the increasing incidence of the disease. The Government of India also evolved a revised strategy and launched the Revised National Tuberculosis Control Programme (RNTCP) based on DOTS strategy in the country in 1997.^2^ Till date RNTCP has put 73,75,867number of patients on treatment and has saved 13,27,656 number of lives.^3^

RNTCP was launched in Chandigarh, a Union Territory (UT) of India, in January 2002. For successful running of any programme, outcome parameters are required to be assessed on continuous basis. Present study aims at studying treatment outcomes for different categories of patients recruited under DOTS in UT Chandigarh.

## MATERIAL AND METHODS

The study was conducted during 2004-2005 in Chandigarh, a Union Territory (UT) of India. Chandigarh has total population of 900,635 with 506,938 males and 393,697 females, as on 31st March 2001.

DOTS is implemented through District Tuberculosis Centre (DTC) in Chandigarh. There are 13 Microscopic centers (MC's) spread over 2 Tuberculosis Units (TU's) under DTC. Under different MC's, a number of DOT centers are functioning. A two-stage stratified random sampling technique was adopted for selection of study subjects. A sample of 8 MC's from both TU's was selected at 1st stage. Patients attending the DOT center for treatment at the first time served as study population. Patients with pulmonary and extra-pulmonary disease, who were above 15 years of age, giving consent to participate in the study, were recruited as study cohort. Informed consent was taken from the respondents and ethical guidelines under Declaration of Helsinki were followed. Institutional Ethical Committee also approved the study. Majority of the patients gave their consent to participate in the study and only a few were reluctant to participate because of their personal problems. A sample of 265 patients was recruited in the study cohort. Information on disease classification, category of treatment, sputum results and other related information was collected during the course of follow-up.

Statistical analysis was carried out using various statistical tests. Normal test of proportions was used for testing significance of difference between proportions. Chi- square test was used for testing association of different characteristics. Kolmogorov-Smirnov (2-sample) test was applied for testing significance of variability between new (Category I and Category III) and re-treatment (Category II) groups. Student's t-test was used for testing significance of difference between means of quantitative parameters. The analysis was carried out using SPSS-10 statistical software package.

## RESULTS

The study included a cohort of 265 patients (231 Pulmonary, 31 Extra-pulmonary). Out of 265 patients enrolled in the present study, 163(61.5%) belonged to Category I, while 52(19.6%) and 50(18.9%) belonged to Category II and Category III respectively (Table 1). Maximum number of the patients belonging to Category I and Category III i.e. 58(35.6%) and 26(52.0%) respectively, were of 15-24 year age group, while that of Category II [18(34.6%)] belonged to 25-34 year age group. The mean age of patients belonging to Category I and Category III were 31.7 + 13.4 years and 32.3 + 14.0 years respectively. For Category II patients, the mean age was 31.7 + 10.1 years. Most of the patients [163(61.5%)] were males, giving a male: female ratio of 1.6:1. Majority of the patients in all the categories were males. Most of the patients were of Hindu religion [233(87.9%)]. The proportion of Category –II patients was significantly higher among Hindus compared to other religion (p<0.001). Most of them [230(86.8%)] belonged to lower socio-economic status. Only one patient belonged to upper class and that to of Category I. Maximum number of the patients i.e. 98(36.9%) were illiterate. Among all the categories also, maximum number of patients were illiterate i.e. 64(39.3%), 17(32.7%) and 17(34.0%) for Category I, II and III respectively.

**Table 1:** Socio-demographic profile of patients by category

Age Group(Yrs)	Category – I No. (%)	Category – II No. (%)	Category – III No. (%)	Total No. (%)	P-Value
15 – 24	58 (35.6)	15 (28.8)	26(52.0)	99 (37.4)	
25 – 34	47 (28.8)	18 (34.6)	9 (18.0)	74 (27.9)	
35 – 44	23 (14.1)	13 (25.)	8 (16.0)	44 (16.6)	P>0.40
45 – 54	15 (9.2)	3 (5.8)	2 (4.0)	20 (7.5)	
55 – 64	16 (9.8)	2 (3.8)	3 (6.0)	21 (7.9)	
>65	4(2.5)	1 (1.9)	2(4.0)	07 (2.6)	
**Mean ± SD (Yrs)**	**31.7 ±13.4**	**32.3 ± 14.0**	**3 1.7 ±10.1**	**29.8 ± 13.8**	
**Sex**					
Male	105 (64.4)	29 (55.8)	29 (58.0)	163 (61.5)	
Female	58 (35.6)	23 (44.2)	21 (42.0)	102 (38.5)	P>0.20
**Religion**					
Hindu	142 (87.1)	49 (94.3)	42 (84.0)	233 (87.9)	
Muslim	9 (3.4)	2(3.8)	4(8.0)	15 (5.7)	P<0.001
Sikh	12 (4.5)	01 (1.9)	4(8.0)	17 (6.4)	
**SES**					
Upper	01 (0.6)	00	01 (0.4)		
Middle	17 (10.4)	5 (9.6)	12 (24.0)	34 (12.8)	P>0.20
Lower	145 (89.0)	47 (90.4)	38 (76.0)	230 (86.8)	
**Education**					
Illiterate	64(39.3)	17 (32.7)	17 (34.0)	98 (36.9)	
Primary school	29 (17.8)	9 (17.3)	4(8.0)	42 (15.9)	
Middle school	29 (17.8)	11 (21.2)	9 (18.0)	49 (18.5)	
High school	25 (15.3)	9 (17.3)	9 (18.0)	43 (16.2)	
Senior secondary	8 (4.9)	5 (9.6)	6 (12.0)	19 (7.2)	P>0.20
Graduate	6(3.7)	1 (1.9)	4(8.0)	11 (4.2)	
PG & Above	2 (1.2)	01 (2.0)	03 (1.1)		

**Total**	**163 (100.0)**	**52 (100.0)**	**50 (100.0)**	**265 (100.0)**	**–**
	**(61.5)**	**(19.6)**	**(18.9)**	**(100.0)**	

Out of 163 patients put on Category I, 146 were New smear positive (NSP). The cure rate among NSP cases was 97.9% and the success rate (cure/treatment completion rate) was 98.6%. Among 52 category II patients (re-treatment group), the overall cure rate was 63.5%, while the success rate was 90.4%. The overall treatment completion rate among 50 Category III patients was 92.0%. The overall default rate in the present study was 1.1%. The default rate was higher in Category II [2(3.8%)] than that found in Category III [1(2.0%)]. None of the patients on Category I defaulted. Failures were maximum in Category II i.e. 3(5.8%), followed by 2(4.0%) in Category III and 2(1.2%) in Category I. The only patient who died belonged to Category III. There was no transferred out case. (Table 2)

**Table 2:** Outcomes by category of treatment

Category	Outcome				
Category–I	Cured No. (%)	Treatment No. (%)	Default No. (%)	Failure No. (%)	Died No. (%)
New smear positive (N=146)	143 (97.9)	01 (0.7)	0	02 (1.4)	0
New smear negative (N=16)	NA*	16 (100.0)	0	0	0
Extra-pulmonary (N=1)	NA*	01 (100.0)	0	0	0
Subtotal (N=163)	143	18 (11.0)	0	02 (1.2)	0

**Category – II**					
Relapse (N=20)	20 (100.0)	0	0	0	0
Failure (N=05)	03 (60.0)	01 (20.0)	01 (20.0)	0	0
TAD** (N=12)	08 (66.7)	0	01 (8.3)	03 (25.0)	0
Others (N=15)	02 (13.3)	13 (86.7)	0	0	0
Subtotal (N=52)	33 (63.5)	14 (26.9)	02 (3.8)	03 (5.8)	0

**Category – III**					
Smear negative (N=25)	NA*	22 (88.0)	01 (4.0)	01 (4.0)	01 (4.0)
Extra-pulmonary (N=25)	NA*	24 (96.0)	0	01 (4.0)	0

**Subtotal (N=50)**	NA*	**46 (92.0)**	**01 (2.0)**	**02 (4.0)**	**01(2.0)**

**Grand Total (N=265)**	**176**	**76**	**03 (1.1)**	**07 (2.6)**	**01(0.4)**

* NA = Not applicable

** TAD = Treatment after default

The sputum conversion rates at 2 months and 3 months among 146 new smear positive (NSP) cases were 88.4% and 93.8% respectively. The overall sputum conversion rate among 34 smear positive re-treatment cases was 94.1% at 3 months of treatment. (Table 3)

**Table 3:** Sputum conversion rates among study cohort

Type of Patient	At 2 months No. (%)	At 3 months No. (%)
**New smear positive (N=146)**	**129 (88.4)**	**137 (93.8)**
**Re-treatment (Smear positive cases)**		
Relapse (N=20)	NA*	20 (100.0)
Failure (N=03)	NA*	01 (33.3)
Treatment after default (N=11)	NA*	11 (100.0)

**Total (N=34)**	NA*	**32 (94.1)**

* NA = Not applicable

## DISCUSSION

In the present study, maximum number of patients belonged to 15-24 year age group. Most of the patients were males, illiterate and belonging to lower socio-economic status. Most of the patients (81.9%) were Hindus, which is slightly higher than the overall proportion of Hindus in the census population (75.8%)^4^. Majority of patients were cured. The failure rate and default rate among Category II patients was high. The treatment completion rate among Category III patients was high. Overall default, failure and death rates were found to be low. The sputum conversion rates, both among new and re-treatment cases were high. However, among the failure cases it was quite low.

The cure/treatment completion rate among NSP and re-treatment patients in the present study was high at 98.6% and 90.4% respectively. The cure rate among NSP cases was high (97.9%). Success rates in terms of cure/treatment completion rates was much higher compared to the national average of 86.0% and 70.5% among the NSP and re-treatment cases respectively.^2^ These rates were also higher compared to those reported by various authors earlier.^5^–^7^

The overall default rate in this study was 1.1%. Majority of patients who defaulted were of Category II i.e. re-treatment cases. The default rate observed in the present study was quite low compared to that reported by other authors.^5^–^7^ The average rate for India was high at 4.0% among NSP and 15.7% among re-treatment cases.^2^

A high failure rate of 5.8% was recorded among patients on Category II, while it was low for Category I (1.2%) and Category III (4.0%) patients. These rates were much lower than that reported earlier^5^ in Delhi. Even lower rates than this were also reported by certain authors.^6^,^7^ The average rate for India was comparable with the present study both for Category I (1.5%) and Category II (5.6%) cases.^2^

None of the patient belonging to Category I and Category II died. The only patient who died belonged to Category III. Earlier authors^5^–^7^ have reported a death rate which was much higher than that reported in the present study. There was no transferred out case recorded in the present study. This finding does not agree with finding of earlier studies.^6^–^7^

The sputum conversion rate among NSP cases at 3 months was 93.8%. Similar high rates have also been recorded by Srivastava SK^6^ and Chadha SL^8^ in their studies. Among the re-treatment cases, the overall conversion rate was 94.1%. This rate was much higher than that reported earlier by various authors.^5^–^6^

The study concludes that RNTCP is running successfully in UT Chandigarh, having high success rate and low default rate. There is an urgent need of sustaining this success.
